# Optimizing cloxacillin prophylaxis in hip and knee arthroplasty based on population pharmacokinetics of unbound plasma concentrations

**DOI:** 10.1093/jac/dkag116

**Published:** 2026-04-03

**Authors:** Gustaf Beijer, Katja Wallander, Bo Söderquist, Christian G Giske, Olof Breuer, Jaran Eriksen, Erik Eliasson

**Affiliations:** Medical Unit of Clinical Pharmacology, MDK, Karolinska University Hospital, Stockholm, Sweden; Division of Clinical Pharmacology, Department of Laboratory Medicine, Karolinska Institutet, Stockholm, Sweden; Department of Infectious Diseases/Venhälsan, Södersjukhuset, Stockholm, Sweden; Department of Clinical Science and Education, Karolinska Institutet, Stockholm, Sweden; Department of Orthopedics, Faculty of Medicine and Health, Örebro University, Örebro, Sweden; School of Medical Sciences, Faculty of Medicine and Health, Örebro University, Örebro, Sweden; Division of Clinical Microbiology, Department of Laboratory Medicine, Karolinska Institutet, Stockholm, Sweden; Medical Unit of Clinical Microbiology, MDK, Karolinska University Hospital, Stockholm, Sweden; Medical Unit of Clinical Pharmacology, MDK, Karolinska University Hospital, Stockholm, Sweden; Division of Clinical Pharmacology, Department of Laboratory Medicine, Karolinska Institutet, Stockholm, Sweden; Department of Infectious Diseases/Venhälsan, Södersjukhuset, Stockholm, Sweden; Department of Clinical Science and Education, Karolinska Institutet, Stockholm, Sweden; Department of Global Public Health, Karolinska Institutet, Stockholm, Sweden; Medical Unit of Clinical Pharmacology, MDK, Karolinska University Hospital, Stockholm, Sweden; Division of Clinical Pharmacology, Department of Laboratory Medicine, Karolinska Institutet, Stockholm, Sweden

## Abstract

**Objectives:**

To characterize the population pharmacokinetics of unbound cloxacillin in patients undergoing total arthroplasty of the hip (THA) or knee (TKA), and to explore alternative dosing regimens for cloxacillin prophylaxis.

**Methods:**

Plasma concentrations of total and unbound cloxacillin from 200 patients undergoing primary elective THA (*n* & 95) or TKA (*n* & 105) were analysed. Intravenous cloxacillin doses of 2 g were administered pre-surgery, and repeated after 2 and 6 hours. Samples (*n* & 496) were analysed using HPLC-MS/MS. Non-linear mixed-effects modelling was performed to develop a population pharmacokinetic model describing unbound cloxacillin exposure. Using this model, alternative prophylaxis regimens were explored with Monte Carlo simulations.

**Results:**

A two-compartment model with non-linear protein binding adequately described the data. Estimated glomerular filtration rate (eGFR) and body weight (kg) were significant covariates on unbound cloxacillin clearance. In 13% of patients sampled at the end of surgery (*n* & 25/187), unbound cloxacillin <2 mg/L was observed. A model-predicted 18–22% (*n* & 36–43/200) of patients failed to sustain plasma levels ≥2 mg/L throughout the two-hour dosing interval with the current regimen. In contrast, a continuous 1 g/h infusion after a 1 g loading dose would ensure target attainment in >99% of patients, according to the model predictions.

**Conclusions:**

For many THA and TKA patients, the current cloxacillin prophylaxis regimen may fail to provide adequate target site concentrations during the entire surgical procedure. Transitioning to a prolonged infusion protocol could increase attainment of PK/PD targets without exceeding the currently recommended total perioperative dose amounts.

## Introduction

Perioperative antibiotic prophylaxis is important to prevent prosthetic joint infections (PJI) after primary arthroplasty of the hip (THA) and knee (TKA).^1^ There is no international consensus regarding agent of choice or the ideal number of doses. Large regional variations exist, partly due to varying MRSA prevalence.^2,3^ In Sweden, cloxacillin is administered in approximately 95% of primary arthroplasty cases.^4^ It has been the first-line option for several decades due to its prominent activity versus methicillin-susceptible staphylococci and its narrow spectrum.^5^ However, the relatively short mean half-life of approximately 30 min^6,7^ necessitates repeat dosing to maintain sufficient systemic concentrations throughout the perioperative period.

Observational data has indicated a higher risk of reoperation for PJI with cloxacillin compared with wider-spectrum alternatives such as e.g. cefazolin.^8^ It is currently unclear to what extent any differences in efficacy might stem from suboptimal cloxacillin dosing regimens. With reported Swedish PJI incidences of 1.2% in the hip^9^ and 1.5% in the knee,^10^ there is probably room for additional improvement.

The current Swedish guidelines recommend an initial 2 g intravenous cloxacillin dose administered over 20–30 min, starting 30–45 min before surgical incision.^11^ An additional 2 g is recommended 2 and 6 h after the first dose, respectively.^11^ This is assumed to ensure high cloxacillin concentrations throughout the perioperative period, with a special emphasis on the time from surgical incision to wound closure.^11,12^ In practice, the second dose is typically administered postoperatively for shorter (<75 min) procedures, and intraoperatively for longer (>75–90 min) procedures.

However, there is limited consensus on the required duration of therapeutic concentrations during the perioperative period. Also, the adequacy of the currently recommended 2 h interval between the first two doses of cloxacillin as prophylaxis in arthroplasty surgery has never been prospectively evaluated. The present study aimed to investigate the pharmacokinetics of unbound cloxacillin in a representative cohort and to estimate the attainment of pharmacokinetic-pharmacodynamic (PK/PD) targets with the current and alternative dosing regimens.

## Materials and methods

### Study design and patient population

This prospective, two-centre study enrolled adult (>18y) patients scheduled for primary THA or TKA between 2022 and 2024. All patients who were non-allergic to penicillin and capable of providing valid consent were eligible for participation. Ethical approval was obtained from the Swedish Ethical Review Authority before commencing recruitment (reference numbers: 2021-02358 and 2024-06520-02). Further details of the study have been previously described.^13^

### Dosing and sampling

Standard doses of 2 g intravenous cloxacillin were administered to all patients at three time points, in accordance with national guidelines.^11^ Infusion durations of 20–30 min were recommended, with the preoperative dose started 30–45 min before surgical incision.^11^ A second dose of 2 g cloxacillin was subsequently recommended 2 h after starting the first dose, followed by a third 2 g dose administered after 6 h.^11^

Blood samples for measuring total and unbound cloxacillin in plasma were collected at three time points per patient: (i) start of surgery, (ii) end of surgery, and (iii) 90 min after the end of surgery, respectively. All samples were collected via separate venipuncture to avoid contamination with drug residues from cloxacillin administration lines. Samples were drawn at the above time points irrespective of when doses were administered.

Only patients with at least one successfully collected blood sample after properly documented start and end times of infusion were included in the population pharmacokinetic analyses.

### Bioanalysis

Blood samples were centrifuged for 10 min at 2000g within 1 h of collection, and the separated plasma was stored at −80°C for up to 6 months pending analysis. Samples were analysed at an accredited university hospital laboratory (the clinical pharmacological laboratory at Karolinska University Hospital, Stockholm, Sweden), where total and unbound cloxacillin plasma concentrations were determined using reversed-phase HPLC-MS/MS methods. Both methods were validated by the laboratory according to EMA guidelines, to the extent that these guidelines were applicable.^14^ Plasma samples demonstrated stability at −80°C for 30 months for both unbound and total cloxacillin. The instruments consisted of a Dionex Ultimate 3000RS LC system coupled to a TSQ Quantis/Quantiva triple quadropole mass spectrometer with an electrospray ionization ion source (Thermo Fisher Scientific, Waltham, MA, USA). Separation of unbound fractions was performed using ultrafiltration according to conditions previously described.^15^ In brief, after preparatory incubation for 30 min at 37°C with 11% ambient CO_2_, 10 min of ultrafiltration at 11 290 g was performed using Sartorius Vivacon 500 (Göttingen, Germany) devices with a 10 kDa molecular weight cut-off membrane.^15^ The analytical range of quantification was 0.1–100 mg/L for total cloxacillin, and 0.01–15 mg/L for unbound cloxacillin.

### Pharmacokinetic modelling

Non-linear mixed effect population pharmacokinetic modelling was performed using Monolix version 2024R1 (Lixoft^®^, Antony, France). One-, two-, and three-compartment model structures were explored. A log-normal distribution of pharmacokinetic parameters was generally assumed. Additive, proportional, and combined additive-proportional error models were explored to describe residual error. Models focusing on only unbound cloxacillin as well as integrated models considering both total and unbound cloxacillin were explored. However, pharmacokinetic parameters were consistently estimated with a specific focus on unbound drug.

Numerous potential covariates affecting the interindividual variability of pharmacokinetic parameters were considered, including age, sex, body weight (total, ideal, and adjusted, respectively), height, BMI, body surface area (BSA), type of prosthesis, operation site, plasma albumin, renal function measures including plasma creatinine, creatinine-based estimated glomerular filtration rate (eGFR) according to CKD-EPI2021,^16^ according to the Lund-Malmö Revised Formula (LMR18),^17–19^ as well as creatinine clearance according to the Cockcroft–Gault equation.^20^ The Du Bois formula^21^ was used to estimate BSA and to convert between relative and absolute GFR estimates. Furthermore, preoperative health status according to the American Society of Anesthesiologists (ASA) classification (I-V), and the type of arthroplasty (hip or knee) were evaluated as potential covariates. For continuous variables, linear as well as logarithmic relationships were tested between individual covariates and model parameters. Covariate selection was based on physiological plausibility, reduction of unexplained parameter variability, improvement of model fit, and statistical significance testing using Pearson’s correlation test for continuous covariates and one-way ANOVA tests for categorical covariates. A stepwise forward inclusion process followed by stepwise backward elimination was used.

The choice between nested models was guided, in part, by objective function value (OFV) reductions of >3.84 (*P* = 0.05). For non-nested models, a reduction of ≥2 points in the Bayesian information criterion (BIC) was generally considered significant.^22^ Basic goodness-of-fit plots, prediction-corrected visual predictive checks (VPC), uncertainty of parameter estimation, and impacts on the correlation matrix of estimates were also considered in all steps of the process to prioritize between models. Non-parametric bootstrap analysis (*n* = 1000) was applied to the final model to verify the robustness of parameter estimates.

### Model-based predictions and simulations

Using the final model developed according to the steps above, concentration-time profiles were predicted based on estimated individual parameter values (empirical Bayes estimates). This way, model-predicted unbound concentrations at specific time points could be evaluated against various *Staphylococcus aureus* MIC targets for every patient in the study population.

In addition, unbound concentrations in virtual patients (*n* = 1000) receiving different prophylaxis regimens were predicted using Monte Carlo simulations. For these simulations, the population parameter estimates from the final model were used, also considering the uncertainty of parameter estimation. Random covariate values for the simulated patients were drawn from distributions that were assumed to be identical to the covariate distributions in our study population.

All simulations were performed using Simulx version 2024R1 (Lixoft^®^, Antony, France). R studio (ver. 2025.05.0 + 496) was used for data management, summary statistics, and graphical presentation of simulation results.

### Target concentrations and definitions of target attainment

The primary target MIC considered was 2 mg/L, with 1 mg/L also of interest as a secondary target. No epidemiological cut-off value (ECOFF) has been established for cloxacillin against *S. aureus* or coagulase-negative staphylococci. However, the structurally similar isoxazolyl penicillin oxacillin has an established ECOFF of 2 mg/L against *S. aureus*,^23^ and a tentative ECOFF of 1 mg/L against *S. aureus* has been suggested for flucloxacillin.^23^ Judging from the single MIC distribution (*n* = 1181) currently available for cloxacillin versus *S. aureus* in the online EUCAST database,^23^ a possible ECOFF of 0.5–1 mg/L seems reasonable to expect.

Since the current prophylaxis guidelines aim to ensure ‘high concentrations’ throughout surgery, unbound cloxacillin plasma concentrations ≥2 mg/L for at least 2 h after starting the preoperative dose were considered compatible with PK/PD target attainment. The 2 h time limit was based on the recommended 2 h interval between the first two doses, combined with the fact that surgery is still ongoing for many patients at the time of the second dose.

## Results

**Figure 1.**
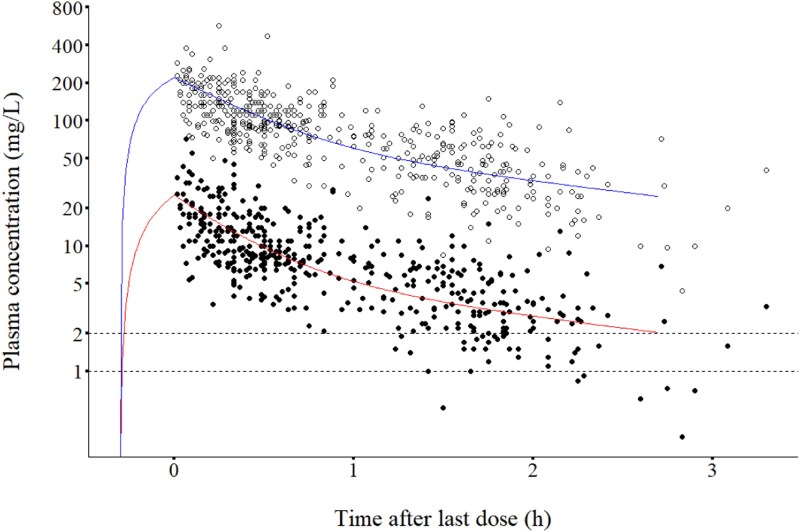
Observed concentrations and typical patient PK profiles according to final model. Observed unbound (filled circles) and total (empty circles) cloxacillin concentrations versus time after dose. The superimposed curves represent model-predicted unbound (red line) and total (blue line) concentrations in a typical patient, with a body weight of 84 kg and eGFR 67 mL/min/1.73 m2 receiving a 20 min infusion of 2 g cloxacillin.

A total of 496 cloxacillin samples from 200 patients (95 THA, 105 TKA) were evaluated. Clinical and demographic characteristics of included patients are presented in Table 1. The median number of samples per patient was 3 (range: 1–3). All observed total and unbound concentrations are shown in relation to time after dosing (Figure 1).

**Table 1.** Patient characteristics and timing of preoperative cloxacillin dose

	Study population (*n* & 200)			
	Count (%)	Median	IQR	Range
Age (years)		73	65–78	36–90
Female sex	105 (53%)			
Body weight (kg)		83	73–95	53–185
BMI (kg/m^2^)		28	25–32	20–61
Plasma creatinine (µmol/L)		70	58–89	11–236
Relative eGFR (mL/min/1.73 m^2^)		72	61–84	18–142
Absolute eGFR (mL/min)		83	68–93	21–158
Plasma albumin (g/L)		34	32–36	15–42
Knee arthroplasty	105 (53%)			
Hip arthroplasty	95 (47%)			
ASA class I	29 (15%)			
ASA class II	84 (42%)			
ASA class III	87 (43%)			
Evaluable samples per time point				
Start of surgery	193 (97%)			
End of surgery	178 (89%)			
90 min postop.	125 (63%)			
Infusion duration (preoperative dose)				
20–30 min^a^	100 (50%)	20	20–25	20–30
<20 min	90 (45%)	15	10–15	0–19
>30 min	10 (5%)	35	33–44	31–60
Start of infusion in relation to incision				
30–45 min before^a^	90 (45%)	39	35–42	30–45
>45 min before	63 (32%)	55	50–61	46–119
<30 min before	47 (23%)	24	21–27	−2.0 to 29
End of infusion in relation to incision				
0–25 min before^a^	114 (57%)	15	9–20	0–25
>25 min before	78 (39%)	33	29–37	26–102
<0 min (ongoing)	8 (4%)	−4	−3 to −9	−1 to −22
No. (%) in accordance with guideline	42 (21%)			

^a^As recommended in current guideline.

The preoperative infusion was administered in accordance with the current guidelines in only 21% (*n* = 42/200) of cases. Shorter-than-recommended infusion durations (<20 min) and earlier-than-recommended administrations in relation to start of surgery were frequently noted (Table 1).

A sample was collected at the start of surgery from 190 patients (95%). The observed median unbound plasma concentration at this time point was 10 mg/L (range: 2.1–55 mg/L).

The corresponding median unbound concentration at the end of surgery was 4.9 (range: 0.3–71) mg/L among the 187 patients (94%) sampled at this time point. Sixty-five (35%) of these patients had received a second dose. Twenty-five patients (13%) had a concentration <2 mg/L, and two patients (1%) had <1 mg/L.

**Figure 2.**
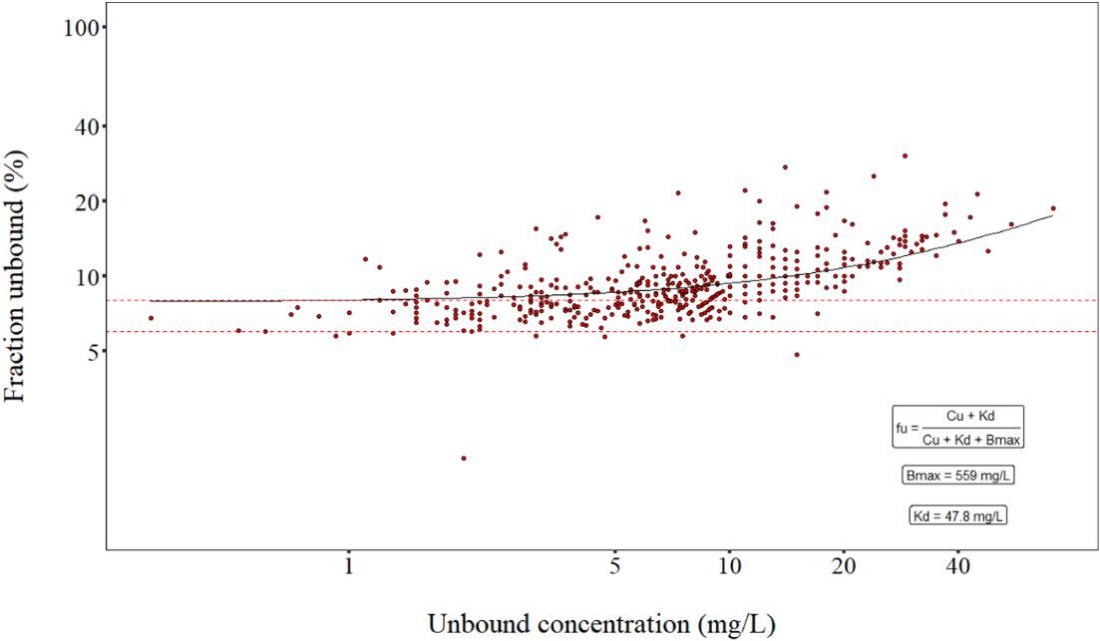
The observed unbound fraction (%) versus unbound cloxacillin concentrations in the study population is plotted. When applying the final model estimates of the dissociation constant (*K_d_*) and the maximum binding capacity (*B*_max_) to the non-linear functions shown in the lower right corner, the black curve (non-linear regression line) is obtained. Red, dashed lines correspond to a reference linear protein binding of 92% and 94%, respectively.

The median observed plasma protein binding was 91% (range: 69–98%), with 98 of 200 patients (49%) displaying a protein binding <90% in at least one sample. Concentration-dependent protein binding was suggested, with an increase in the unbound fraction at high concentrations (Figure 2).

### Population pharmacokinetic model development

A two-compartment model with linear elimination and non-linear protein binding (Table S2, available as data at *JAC* Online, appendix, Figure S1) described the data well.

Relative eGFR_LMR18_ and total body weight (BW) were introduced as CL covariates, after verifying their respective improvement of model fits. Goodness-of-fit plots, residual diagnostics, and visual predictive checks generally indicated an adequate predictive performance (Figures S2–S4). For additional model details, please refer to the Supplementary material. In Figure 1, model-predicted concentration-time profiles of total and unbound cloxacillin for a typical patient are superimposed on the observed concentrations, for visual reference.

### Model-predicted target attainment in the study population

**Figure 3.**
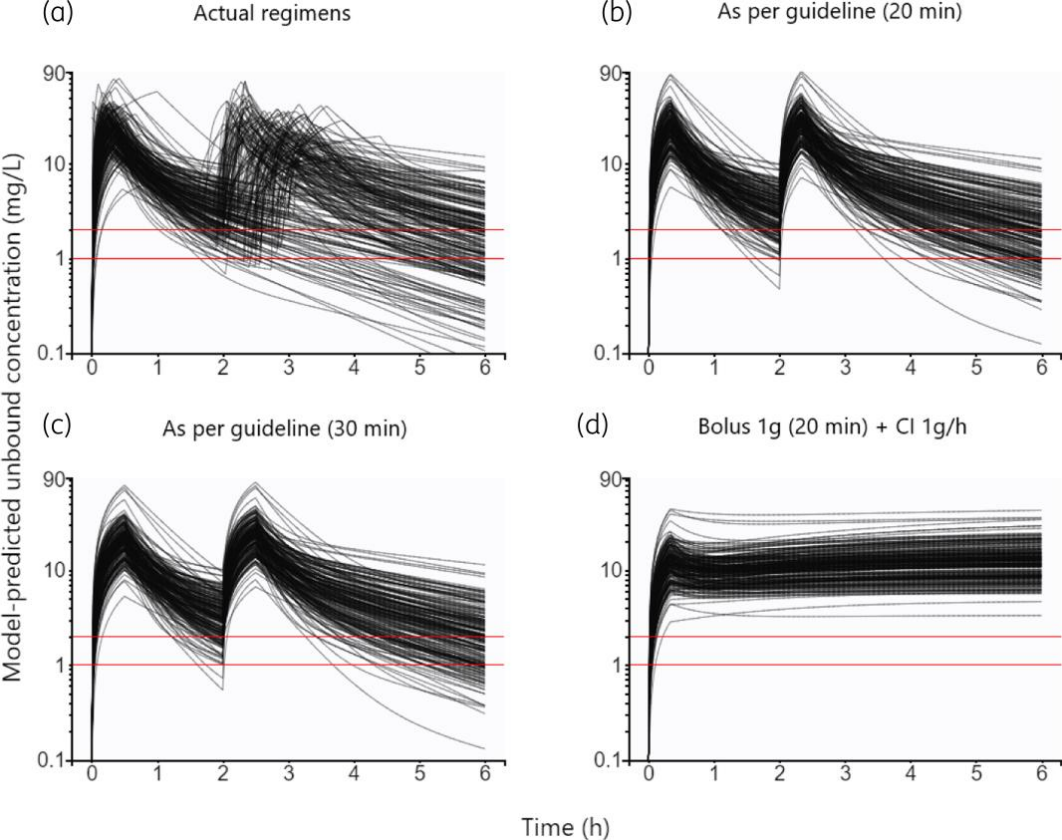
Model-predicted concentration-time profiles in the study population (*n* = 200). Predicted concentration-time profiles in the study population, based on documented infusion times and dosing occasions (a). Model-predicted profiles for the same patients receiving 20 min (b) and 30 min (c) 2 g infusions with the recommended 2 h interval are also shown, in addition to predicted profiles after a 1 g loading dose followed by a 1 g/h continuous infusion (d). The red lines correspond to the target concentrations of 2 mg/L and 1 mg/L, respectively.

The individual concentration–time profiles, as predicted by the final model, illustrate the variable infusion durations and administration times noted in the study population (Figure 3a). According to the model predictions, the median unbound concentration 2 h after starting the preoperative dose was 3.2 (range: 0.7–42) mg/L. Importantly, 18 patients (9%) had received a second 2 g dose at this time (Figure 3a). Excluding these patients, the median 2 h concentration was 3.1 (range: 0.7–14) mg/L. Thirty-seven patients (19%) had a model-predicted unbound concentration <2 mg/L two hours after starting the preoperative infusion, and 3% (*n* & 5/200) had a model-predicted 2 h concentration <1 mg/L.

If all patients had received the preoperative dose over 20 min (Figure 3b), a model-predicted 22% (*n* & 43/200) and 3% (*n* & 5/200) would have had unbound concentrations below 2 mg/L and 1 mg/L two hours after infusion start, respectively, assuming adherence to the recommended 2 h dosing interval. With a 30-min infusion, the corresponding proportions would have been 18% (*n* & 36/200) and 1% (*n* & 2/200), respectively, according to the model predictions (Figure 3c).

In contrast, if all patients had received a 1 g loading dose over 20 min, followed by a continuous infusion of 1 g/h (Figure 3d), the entire study population would have stayed above both targets for the duration of infusion, according to the model predictions (Figure 3d). Two hours after the start of such a regimen, a median unbound concentration of 10.5 (range: 3.3–38.6) mg/L was predicted.

### Model-predicted target attainment in simulated patients

**Figure 4.**
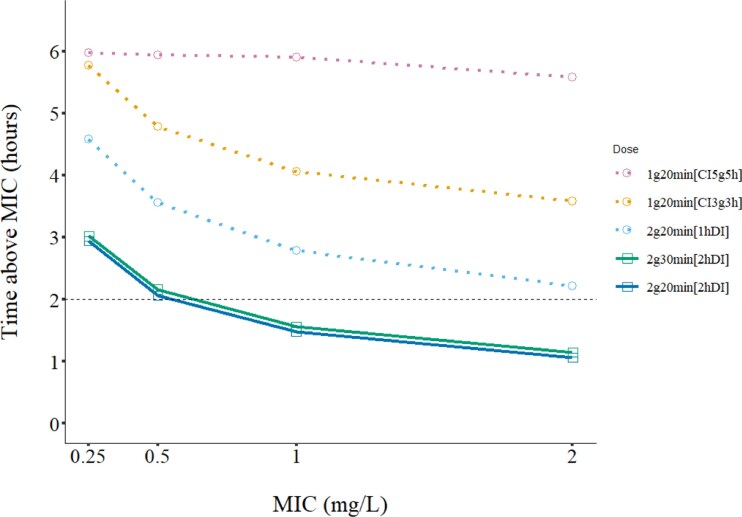
Model-predicted time above MIC with various prophylaxis regimens. The plot shows the model-predicted time (hours) during which unbound cloxacillin concentrations in plasma remain above MIC in 99% of simulated individuals (*n* = 1000). Each line represents a distinct dosing regimen, with points connected across the four evaluated MIC levels. The annotation 1g20 min[CI5g5h] corresponds to a 20 min infusion of 1g followed by a continuous infusion (CI) of 5 g over 5 h. In contrast, 2g20 min[1hDI] denotes a 20 min infusion of 2 g followed by another 20 min infusion of 2 g after 1 h. The dashed black line corresponds to the currently recommended time interval (2 h) between the first two doses. With the currently recommended 2g30 min[2hDI] and 2g20 min[2hDI] regimens (highlighted, green and dark blue lines), unbound cloxacillin is predicted to decline to <2 mg/L in approximately 25% of patients before the second dose, according to the model-based simulations.

Beyond the observed study cohort, model-predicted time above MIC was estimated for various intermittent and continuous regimens based on the final population model (Figure 4).

Among 1000 simulated patients receiving 2 g cloxacillin over 20 min, a model-predicted 26% (*n* & 255) showed unbound concentrations <2 mg/L within 2 h after infusion start, and 6% (*n* & 61) had <1 mg/L. With a 30 min infusion time, the corresponding proportions were 24% (*n* & 238) and 5% (*n* & 51), respectively.

To ensure unbound plasma concentrations >1–2 mg/L for at least 2 h in ≥99% of patients, alternative regimens would be required according to the model-based predictions (Figure 4).

One option could be an extended or continuous infusion after administering a 1–2 g loading dose. According to the model predictions, starting a 1 g/h continuous infusion after a 1–2 g loading dose should ensure sufficient unbound plasma concentrations at least for the duration of infusion in >99% of patients (Figure 4).

## Discussion

The findings of the present study challenge the adequacy of the currently recommended cloxacillin prophylaxis regimen for patients undergoing THA or TKA. During the 2-h interval between initial doses, a substantial proportion of patients failed to sustain unbound plasma concentrations above reasonable MIC targets, according to our findings. Consequently, sufficient exposure levels to prevent PJI cannot be assumed.

Our findings may at least partly explain the higher risk of reoperation for PJI reported with cloxacillin compared with the common cephalosporin prophylaxis alternatives cefazolin and cefuroxime.^8^ With typical half-lives more than twice as long as cloxacillin,^24,25^ timing of the second dose may be less critical for these drugs. Based on the findings of the present study, we argue that intermittent cloxacillin prophylaxis is no longer justifiable. Switching to a cephalosporin or optimizing the administration of cloxacillin is likely warranted.

Our simulations, based on the unbound pharmacokinetics in a large cohort of arthroplasty patients, provide insight into how the present cloxacillin regimen may be refined. Although body weight and eGFR showed significant correlations with the clearance of unbound cloxacillin, they explained only a limited part of the observed inter-individual variability in achieved plasma concentrations. Thus, our findings suggest that individualized dosing based solely on anthropometric variables and plasma creatinine-based estimates of renal function is probably not sufficient to ensure target attainment in an acceptable proportion of patients.

Furthermore, surprisingly few patients received the preoperative dose in accordance with recommendations. Similarly, disappointing guideline adherence has been previously reported,^4,26^ which indicates that the problem is not unique to the centres in the present study. An important reason might be the unpredictable nature of clinical practice, where anaesthetic and surgical timing variability hampers consistent adherence to recommended dosing intervals. In addition, the use of conventional gravity infusion sets is likely to increase the variability in infusion durations compared with pump-driven infusions.

Transitioning to a pump-driven continuous infusion of 1 g cloxacillin per hour after an initial loading dose of 1–2 g would probably increase cloxacillin target attainment throughout surgery, according to our findings. The total dose of cloxacillin administered with such an approach would not necessarily exceed the currently recommended 6 g. An increased ease of administration and enhanced protocol adherence should be expected with a regimen that relies on pre-programmed infusion pumps. During the preoperative time-out, an ongoing cloxacillin infusion would also be a more definitive verification to all team members that antibiotic prophylaxis has not been prematurely administered or even forgotten.

The observed median plasma protein binding in our population was slightly lower than the 92–94%^6,27^ commonly reported by cloxacillin manufacturers. This was not surprising since protein binding information in cloxacillin monographs is probably based on data from young, healthy volunteers with higher levels of plasma albumin and better-preserved renal function than average THA and TKA patients. Indeed, previous studies have generally reported lower levels of cloxacillin protein binding in various real-life patient populations.^28,29^ Our findings add to this body of knowledge and provide further insight into the wide variability in cloxacillin protein binding (69–98%) encountered in clinical practice. This is important to consider when interpreting TDM results based on total plasma concentrations adjusted for assumed protein binding.

### Strengths and limitations

The direct measurement of unbound plasma concentrations and the large number of enrolled patients were major strengths. In addition, the variable sampling time points, dictated by surgical events rather than cloxacillin dosing, ensured good coverage of the entire post-dose concentration time course. This was beneficial from a modelling perspective and increased confidence in parameter estimations. Furthermore, both sexes were well-represented, and the study population was diverse in terms of age, body size, and preoperative health status.

An obvious limitation was the reliance on plasma measurements rather than direct bone tissue measurements. However, due to unresolved methodological issues associated with measuring bone tissue concentrations in a reliable manner, and the uncertainties regarding which exact compartments would be of primary interest in the context of PJI, we believe that our focus on unbound plasma concentrations was reasonable. Another limitation was the frequently incomplete information available regarding, e.g. start or end times of cloxacillin infusions, which led to a relatively large proportion of missing samples for population pharmacokinetic analyses.

Plasma albumin levels and eGFR were generally relatively well preserved in the study population, with few patients displaying very low levels. This limits the possibility to draw conclusions regarding for example exposure in patients with severe renal impairment.

In addition, since the cloxacillin MIC distribution among wild type *S. aureus* isolates remain undefined, the suggested MIC targets and specific PK/PD target attainment rates in the present study should be interpreted with caution.

### Future perspectives

Considering the low incidence of PJI in modern practice, prospective studies with clinical endpoints comparing standard intermittent dosing versus continuous prophylaxis regimens would need to enrol many patients to demonstrate any significant differences. In Sweden and comparable countries, automatic randomization via existing quality registers could be a feasible approach to consider from a logistical and economic perspective. By seeking to optimize current dosing practices, narrow-spectrum penicillins might remain a viable option to wider-spectrum alternatives. This way, reduced rates of PJI could potentially be achieved without negatively impacting antimicrobial resistance development.

### Conclusion

For many THA and TKA patients, the current cloxacillin prophylaxis regimen may fail to provide adequate target site concentrations during the entire surgical procedure. Transitioning to a prolonged infusion protocol could increase attainment of PK/PD targets without exceeding the currently recommended total perioperative dose amounts.

## Supplementary Material

dkag116_Supplementary_Data

## Supplementary data

Figures S1 to S4, Table S2 and Appendices are available as Supplementary data at *JAC* Online.
