# Supplementary material for: Optimizing cloxacillin prophylaxis in hip and knee arthroplasty based on population pharmacokinetics of unbound plasma concentrations
Source: J Antimicrob Chemother. 2026 Apr 3;81(5):dkag116. doi: 10.1093/jac/dkag116 (PMC13049546; doi:10.1093/jac/dkag116)
Supplement: dkag116_Supplementary_Data [file dkag116_supplementary_data.docx]

**Supplementary material**

**Population pharmacokinetic model development details**

A log-additive residual error model was found to be appropriate for both unbound and total concentrations. A non-linear one site-specific protein binding model was used to describe the relationship between total and unbound concentrations. Interindividual variability could be estimated for unbound clearance (CL), central volume of distribution (V1), intercompartmental clearance (Q), and the maximum protein binding capacity in plasma (Bmax). The interindividual variability for CL and V1 were significantly positively correlated. Thus, correlation (ρ) was introduced as a separate model parameter, calculated by dividing the estimated covariance between variables with the product of the respective standard deviations (ω) of their random effects (see separate section below).

Of the various plasma creatinine-based eGFR equations evaluated, eGFR_LMR18_ showed the strongest positive correlation with interindividual variability (η_CL_) in CL (Table S1). In the final model, relative eGFR_LMR18_ and total body weight (BW) were both retained as significant CL covariates, after verifying their respective improvement of model fits. Unexplained interindividual variability in CL was reduced from 59% to 50% after introducing the eGFR_LMR18_ covariate, and from 50% to 47% after including body weight. No other covariates were found to significantly improve the model fit.

Although plasma albumin levels were significantly correlated with protein binding, adding it as a covariate on B_max_ (and K_d_) led to over-fitting and did not improve overall model performance. Thus, plasma albumin was not retained as a covariate in the final model.

All parameters of the final model were estimated with acceptable precision (see Table S2). On an individual level, relatively high EBE η-shrinkage was observed for Bmax (77%) and Q (37%), compared to V1 (22%) and CL (11%). In contrast, the corresponding η-shrinkages were <3% for all parameters when sampled from their respective conditional distributions, in accordance with the Monolix default option for increasing reliability of diagnostic plots. The ε-shrinkage for the residual error of unbound concentrations was 19.5%.

**Correlation between parameters**

In accordance with the default option in Monolix for handling correlations between random effects,^1^ the correlation (*ρ*) between clearance (CL) and the central volume of distribution (V_1_) was introduced as a separate parameter in the final model, estimated according to the relationship:

$\rho_{(CL, V_{1})}= \frac{covar(Cl, V_{1})}{\sqrt{var(CL)} x \sqrt{var(V_{1})}}$

which corresponds to :

$\rho_{(CL, V_{1})}= \frac{\omega_{CL,V_{1}}}{\omega_{CL} x \omega_{V_{1}}}$

when considering the Ω matrix (with respect to only CL and V_1_):

$\Omega=\left[ \begin{matrix} \omega_{CL}^{2} & \\ \omega_{CL,V_{1}} & \omega_{V_{1}}^{2} \end{matrix} \right]$

**Protein binding**

A non-linear relationship between total and unbound concentrations could be adequately described with a one site-specific binding base model according to the equation:

$Ctot = Cu + \frac{Bmax \times C_{u}}{K_{d}+ C_{u}}$ *(1)*

In this equation, *C_tot_ = total cloxacillin (mg/L), C_u_* = unbound cloxacillin (mg/L), *Bmax* = maximum binding capacity (mg/L), and *Kd* is the dissociation constant.^2^

After verifying that the above non-linear function seemed to adequately describe the relationship between total and unbound cloxacillin concentrations in our study population, B_max_ and K_d_ were introduced as separate parameters to be estimated in the population pharmacokinetic model.

**Renal function covariate selection**

Initially, the LMR18_abs_ eGFR estimate, with and without log-transformation (Table S1), was evaluated as a CL covariate. However, various anthropometric covariates (adjusted/ideal/total body weight, BMI, body surface area) remained independently correlated with model-predicted CL after such inclusion, at a high significance level (p<0.001). To reduce collinearity issues, relative eGFR (LMR18_rel_) and body weight were evaluated in parallel as separate CL covariates.

Comparable OFV and AIC/BIC reductions were seen after introducing logt(eGFR-LMR18_abs_) as a CL covariate (OFV: -42, AIC: -40, BIC: -37, BICc: -37), compared with the combined introduction of logt(eGFR-LMR18_rel_) and logt(WEIGHT) as separate CL covariates (OFV: -49, AIC: -45, BIC: -38, BICc: -38). The latter option was chosen, based primarily on visual predictive checks (pcVPC), increased confidence in parameter estimates, and significant reductions of OFV/AIC.

Table S1. Evaluated GFR equations and their respective correlation with η_CL_

|  | **Correlation coefficient** | **Statistic**  **(t)** | **P-value** |
| --- | --- | --- | --- |
| CKDEPI-2021_rel_ | 0.15 | 2.07 | 3.97e-2 |
| Logt(CKDEPI-2021_rel_) | 0.17 | 2.38 | 1.8e-2 |
| CKDEPI-2121_abs_ | 0.29 | 4.31 | 2.53e-5 |
| Logt(CKDEPI-2121_abs_) | 0.30 | 4.38 | 1.91e-5 |
| CrCL_original_ (C-G) | 0.21 | 2.96 | 3.49e-3 |
| Logt(CrCL_original_ (C-G)) | 0.32 | 4.80 | 3.11e-6 |
| CrCL_weight-adjusted_ | 0.18 | 2.56 | 1.13e-2 |
| Logt(CrCL_weight-adjusted_) | 0.30 | 4.37 | 1.99e-5 |
| LMR18_rel_ | 0.32 | 4.68 | 5.35e-6 |
| Logt(LMR18_rel_) | 0.33 | 4.93 | 1.70e-6 |
| LMR18_abs_ | 0.43 | 6.79 | 1.28e-10 |
| Logt(LMR18_abs_) | 0.44 | 6.83 | 1.03e-10 |

Logt=Log-transformed (see below)

Table S2. Parameters of the final population pharmacokinetic model for unbound cloxacillin

|  | |  |  | **Bootstrap** |
| --- | --- | --- | --- | --- |
| **Parameter** | | **Population estimate** | **RSE (%)** | **median (95% CI)** |
|  | |  |  |  |
| **CL (L/h)** = $\mathbf{ϴ}_{\mathbf{CL}} \mathbf{x} \left( \frac{\mathbf{BW}}{\mathbf{84}} \right)^{\boldsymbol{\beta}_{\mathbf{1}}} \mathbf{x} \left( \frac{\mathbf{eGFR}}{\mathbf{67}} \right)^{\boldsymbol{\beta}_{\mathbf{2}}} \mathbf{x}\mathbf{e}^{\boldsymbol{\eta}_{\boldsymbol{CL}}}$ | |  |  |  |
|  | ϴ_CL_ | 76.4 | 4.4 | 74.3 (61.4 – 83.3) |
|  | β_1_ | 0.67 | 22 | 0.71 (0.39 – 1.07) |
|  | β_2_ | 0.51 | 18 | 0.53 (0.33 – 0.75) |
|  | ω_CL_ (CV) | 0.45 (47%) | 7.1 | 0.44 (0.36 – 0.55) |
|  | |  |  |  |
| **V_1_ (L) =**$\mathbf{ϴ}_{\mathbf{V1}} \mathbf{x}\mathbf{e}^{\boldsymbol{\eta}_{\boldsymbol{V}\boldsymbol{1}}}$ | |  |  |  |
|  | ϴ_V1_ | 57.1 | 7.7 | 56.5 (43.8 – 70.8) |
|  | ω_V1_ (CV) | 0.64 (72%) | 9.2 | 0.64 (0.50 – 0.76) |
|  | |  |  |  |
| **Q (L/h) =** $\mathbf{ϴ}_{\mathbf{Q}}$ $\mathbf{x} \mathbf{e}^{\boldsymbol{\eta}_{\boldsymbol{Q}}}$ | |  |  |  |
|  | ϴ_Q_ | 57.2 | 12 | 56.1 (42.6 – 96.3) |
|  | ω_Q_ (CV) | 0.79 (94%) | 13 | 0.77 (0.49 – 1.19) |
|  | |  |  |  |
| **V_2_ (L) =** $\mathbf{ϴ}_{\mathbf{V2}}$ | |  |  |  |
|  | ϴ_V2_ | 69.0 | 10 | 72.3 (53.4 - 153) |
|  | |  |  |  |
| **B**_max_ **(mg/L) =** $\mathbf{ϴ}_{\mathbf{B}_{\boldsymbol{max}}}\mathbf{x} \boldsymbol{e}^{\boldsymbol{\eta}_{\boldsymbol{Bmax}}}$ | |  |  |  |
|  | ϴ_Bmax_ | 559 | 1.6 | 564 (511 - 625) |
|  | ω_Bmax_ (CV) | 0.05 (4.7%) | 25 | 0.05 (0.03 – 0.10) |
|  | |  |  |  |
| **Kd (mg/L) =** $\mathbf{ϴ}_{\mathbf{K}_{\boldsymbol{d}}}$ | |  |  |  |
|  | ϴ_Kd_ | 47.8 | 2.6 | 48.7 (43.0 – 55.0) |
|  | |  |  |  |
| **ρ_CL~V1_** | | 0.68 | 9.8 | 0.67 (0.50 – 0.78) |
|  | |  |  |  |
| **log(Y) = log(f) + a** x **ε** | |  |  |  |
|  | a_(unbound)_ | 0.24 | 4.4 | 0.24 (0.20 – 0.27) |
|  | a_(total)_ | 0.19 | 4.6 | 0.19 (0.16 – 0.22) |

BW=Total body weight. CL=Clearance. CV=Coefficient of variation. eGFR=Estimated glomerular filtration rate. V_1_=Central volume of distribution. V_2_=Peripheral volume of distribution. Q=Intercompartmental clearance. B_max_=Maximum protein binding capacity parameter. K_d_=Protein binding dissociation parameter. ρ_CL~V1_=Correlation parameter (CL vs. V_1_). log(Y)=Observed data (log-transformed). log(f)=Function describing the structural model. ϴ=Typical population parameter value. β=Covariate coefficient (exponent). ω=Standard deviation of inter-individual parameter variability (IIV). RSE=Residual standard error.

**Figure S1. Structural illustration of the pharmacokinetic model and its key parameters.**
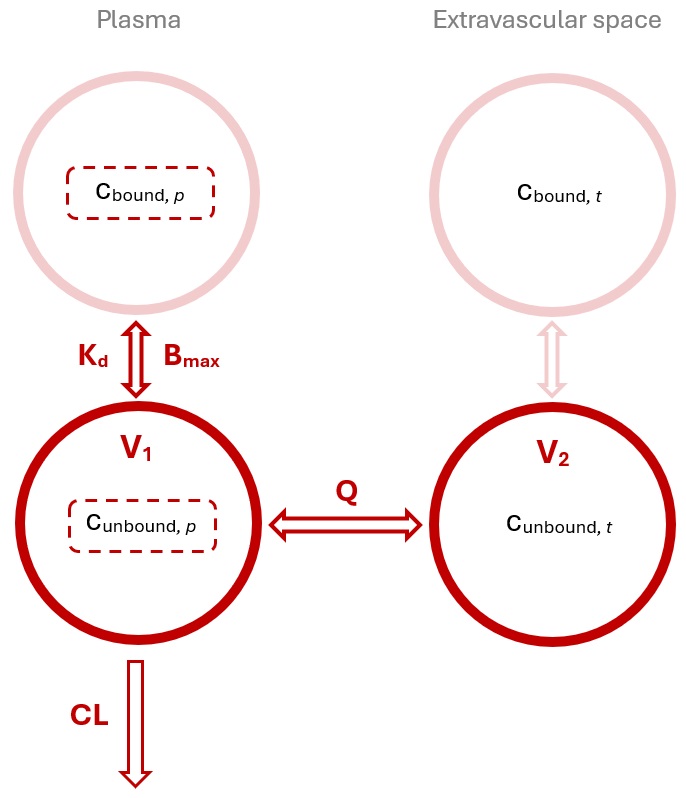


Cunbound, p = Unbound plasma concentration (measured in the present study, mg/L). Cbound, p =Bound plasma concentration (corresponding to total minus unbound concentration). C(un)bound, t = (Un)bound tissue concentration (not measured in the present study). Bmax=Maximum binding parameter. CL=Clearance (L/h). Kd=Dissociation constant. Q=Intercompartmental clearance (L/h). V1=Central volume of distribution. V2=Peripheral volume of distribution.

**Figure S2. Prediction corrected visual predictive checks (pcVPC).**
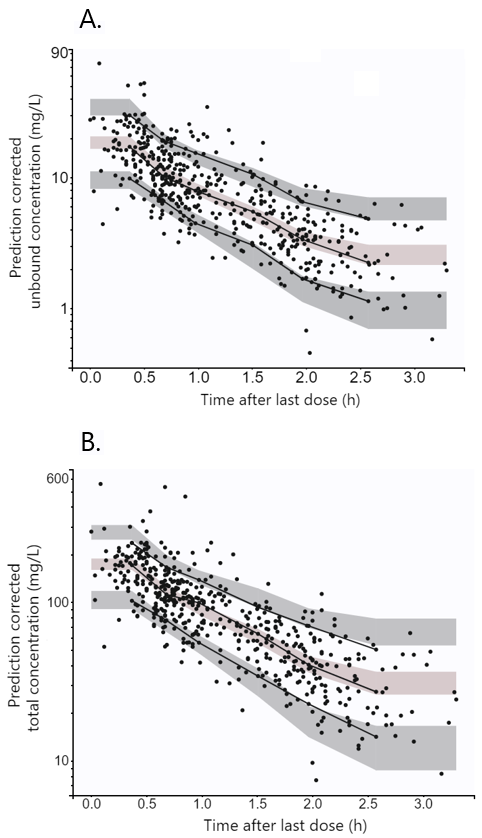


**Figure S3. Observations versus predictions of unbound and total cloxacillin**


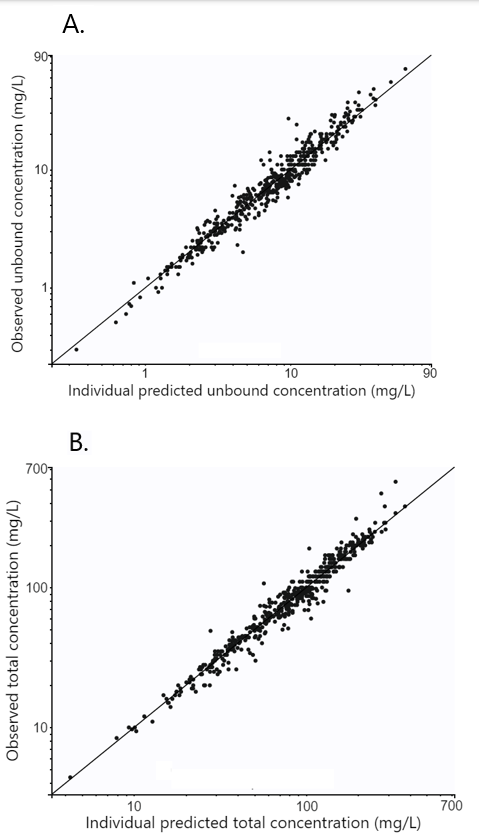


**
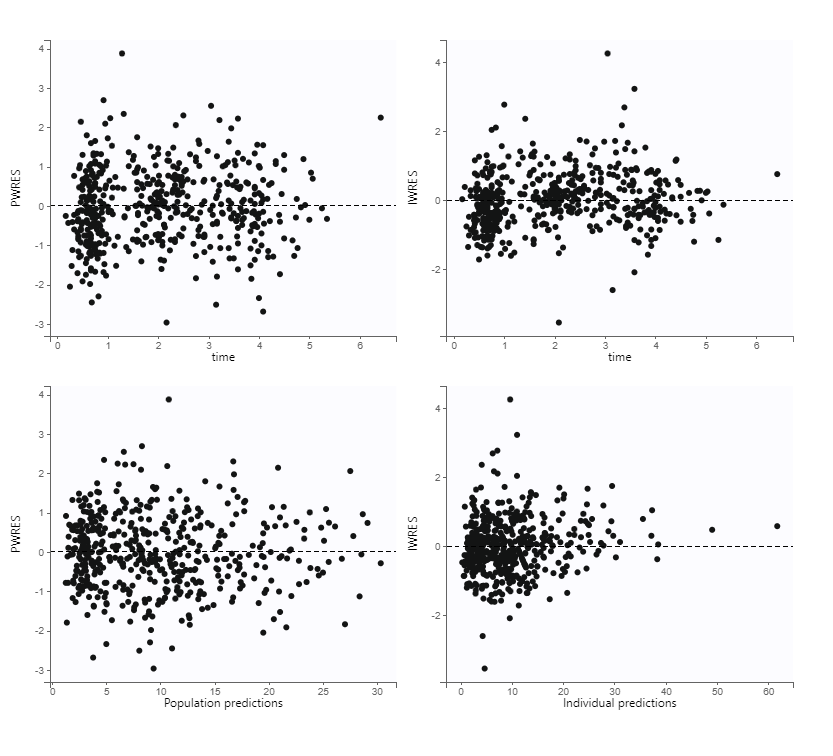
Figure S4. Population (PWRES) and individual weighted residuals (IWRS) plots**

**References**

1. Lixoft documentation. Distribution of individual parameters. Correlation structure of the random effects. Available via: monolixsuite.slp-software.com/monolix/2024R1
2. Toutain et al. Free drug fraction vs. free drug concentration: a matter of frequent confusion. J Vet Pharmacol Ther. 2002 Dec;25(6):460-3.

**
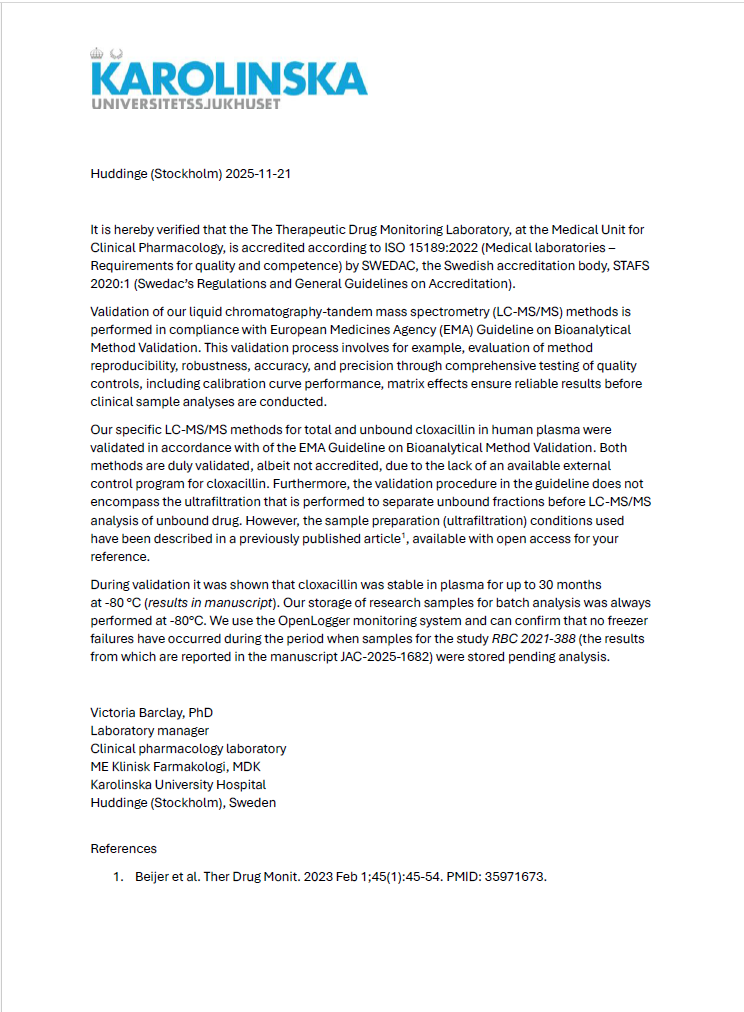
S5. Bioanalytical method validation statement**
